# Editorial: Women in psychiatry: personality disorders 2023

**DOI:** 10.3389/fpsyt.2024.1420591

**Published:** 2024-05-16

**Authors:** Nadine Larivière, Suzane Renaud, Francesca Strappini

**Affiliations:** ^1^ École de réadaptation, Université de Sherbrooke, Sherbrooke, QC, Canada; ^2^ Centre intégré de santé et services sociaux (CISSS) des Laurentides, St. Jerome, QC, Canada; ^3^ Department of Philosophy and Communication Studies, University of Bologna, Bologna, Italy

**Keywords:** personality disorders (PDs), alternative model for personality disorders (AMPD), interpersonal functioning, therapeutic interventions, intrapersonal personality dynamics

Looking back more than four decades, the field of research in personality disorders (PD) has come a long way attaining theoretical and practical understanding of the concept, particularly with the shift from a categorical approach to a dimensional one, supporting both biological and psychological studies. The clinical picture about comorbidity, evolution, and prognosis has been enhanced by longitudinal studies, laboratory research, and neuroimaging. Many psychotherapeutic theories and techniques have been developed and tested, bringing hope to those suffering from PD.

Foremost, psychiatric research methodologies have greatly improved, and this Research Topic of Frontiers in Psychiatry: Personality Disorders Research bears witness to this progress, emphasizing the significant contribution of scientific women as leaders of research teams and authors. Engaged in investigating the lived experience of persons with PD, this Research Topic shows that their current topics of interest tap into the potential of the alternative dimensional model of PD to closely examine interpersonal functioning. In addition, interestingly, this Research Topic not only concerns people with borderline personality disorder but also pathological narcissism and paranoid personality disorder. The overall findings described in the papers enrich our understanding of the manifestation of personality traits and symptoms in men and women to better inform current and future treatments and interventions.

Personality and gender are intimately linked and influence establishment of diagnoses. Therefore, comparison between women and men are important to investigate. Bozzatello et al. conducted a narrative review to summarize current state of knowledge regarding similarities and differences between men and women with BPD regarding their temperamental and clinical characteristics, comorbid conditions, brain mechanisms and activation, as well as service and treatment utilization. A key message is that although there are many commonalities in the clinical presentation of BPD between women and men, distinctions exist in several areas, and these need to be taken into consideration when assessing and treating without bias.

Comparing persons with lived experience of PD to adults from the community or recruited in different clinical settings on features influencing intrapersonal functioning and interpersonal functioning, provide valuable information on targets and strategies for effective therapeutic approaches. Relationship satisfaction is an essential outcome that challenges persons living with PDs. Savard et al. examined couple satisfaction and dissatisfaction though the lens of the alternative model of the DSM-5. Their findings show the attachment style of persons with PD is activated when engaging in an intimate relationship. Consequently, intimacy avoidance and separation insecurity emerge as significant predictors.

An essential concept that affects social functioning is the construct of theory of mind, which has not been extensively investigated in PD. It was explored by Lampron et al. through clustering of profiles with varying level of theory of minds skills in adults with PD and others recruited form the community. Personality traits of antagonism and detachment (to a lesser degree) were found to be associated with theory of mind dysfunctions.

People with paranoid personality disorder are also challenged in their social functioning and have their own ways of composing with the world. Jacobs proposes a philosophical point of view to incorporate the concept of *oikeiosis*, related to notions of feelings of familiarity, belonging and trustful relatedness to oneself and others. This concept also considers changes in intuition characteristic of paranoia that influence *oikeiosis*. Jacobs suggests adopting a holistic view of the paranoia experience in introspective approaches to activate resilience factors for an existential repositioning of trust, familiarity, and social belonging.

Negative events that induce a negative mood can affect anyone with PD, but how does someone with pathological narcissism try to feel better in those circumstances? According to Finch and Hooley in their study, grandiose fantasizing is consciously chosen as a negative affect regulation strategy in the short term in those who present a combination of grandiose and vulnerable narcissistic traits. The scenarios that color grandiose fantasies tend to have low plausibility, high ambitiousness, and high agency compared to positive future thinking. This paves the way to stimulating future avenues.

People with PD often present other mental health issues, as reflected in the findings of the study by Lunghi et al. Among therapeutic options, medication use is one component that merits attention. The Quebec descriptive study on psychotropic medication in cluster B personality disorder patients, through a large population healthcare registry has the merit of examining what occurs beyond practice guidelines that do not recommend drugs as solutions to PDs or clarifying patients’ needs affected by significant associated comorbidities. The study reveals a surge of prescription around the time of diagnosis, indicating a critical phase, although the underlying issues persist even after diagnosis. The article hints at the comorbidities potentially explaining the tendencies to prescribe antidepressants, antipsychotics and stimulants, but the presence of the coded anxious-depressive diagnosis in half the cases does not provide a complete picture. Interestingly, social deprivation was not a factor of overprescribing, whereas social advantage appears to influence doctors’ prescription habits. This suggests that social status can influence medical decisions regarding medication.

Research investigations about PD are also delving into understanding the mechanisms behind psychotherapeutic interventions and their impact. This demonstrates an advancement in our comprehension of psychotherapy, which is a principal intervention for PDs. Buronfosse et al. tested the effectiveness of adding a dialectical behavioral therapy-based telephone hotline provided by psychologists for patients with borderline personality disorder. They aimed to examine its effectiveness in reducing suicidal attempts and self-injurious behaviors, compared to treatment as usual, across clinical samples from nine centers in France. The analysis demonstrated that the hotline reduced the suicide risk by 70% and self-injurious behavior by 83%, after adjusting for several variables. Although only around one-third of the participants used the hotline, most reported feeling that its availability offered some relief. The authors argue that such support is easy to use, cost-effective, and easy to implement.

To conclude, women have always been important contributors in research on PDs and this Research Topic is a statement to their dynamism in providing rigorous and influential information in a field that has greatly expanded in both its topics and research methodologies.

## Author contributions

NL: Writing – original draft, Writing – review & editing. SR: Writing – original draft, Writing – review & editing. FS: Writing – review & editing.

